# Metabolomics-based analysis of miniature flask contents identifies tobacco mixture use among the ancient Maya

**DOI:** 10.1038/s41598-021-81158-y

**Published:** 2021-01-15

**Authors:** Mario Zimmermann, Korey J. Brownstein, Luis Pantoja Díaz, Iliana Ancona Aragón, Scott Hutson, Barry Kidder, Shannon Tushingham, David R. Gang

**Affiliations:** 1 Department of Anthropology, Washington State University, P.O. Box 644910, Pullman, WA 99164-4910 USA; 2 Centro Regional Yucatán, National Institute of Anthropology and History, Col. Gonzalo Guerrero, Calle 10 #310-A, 97310 Mérida, Yucatán Mexico; 3 Department of Anthropology, University of Kentucky, 211 Lafferty Hall, Lexington, KY 40506-0027 USA; 4 Institute of Biological Chemistry, Washington State University, P.O. Box 646340, Pullman, WA 99164-6340 USA; 5 Present Address: Department of Molecular Genetics and Cell Biology, The University of Chicago, Cummings Life Science Center, 920 E. 58th Street, Suite 1106, Chicago, IL 60637 USA

**Keywords:** Molecular biology, Environmental social sciences

## Abstract

A particular type of miniature ceramic vessel locally known as “veneneras” is occasionally found during archaeological excavations in the Maya Area. To date, only one study of a collection of such containers successfully identified organic residues through coupled chromatography–mass spectrometry methods. That study identified traces of nicotine likely associated with tobacco. Here we present a more complete picture by analyzing a suite of possible complementary ingredients in tobacco mixtures across a collection of 14 miniature vessels. The collection includes four different vessel forms and allows for the comparison of specimens which had previously formed part of museum exhibitions with recently excavated, untreated containers. Archaeological samples were compared with fresh as well as cured reference materials from two different species of tobacco (*Nicotiana tabacum* and *N. rustica*). In addition, we sampled six more plants which are linked to mind-altering practices through Mesoamerican ethnohistoric or ethnographic records. Analyses were conducted using UPLC-MS metabolomics-based analytical techniques, which significantly expand the possible detection of chemical compounds compared to previous biomarker-focused studies. Results include the detection of more than 9000 residual chemical features. We trace, for the first time, the presence of Mexican marigold (*Tagetes lucida*) in presumptive polydrug mixtures.

## Introduction

The induction of altered states of consciousness (ASC) is a common feature of humankind^1^, among hunting and gathering communities^2^ as well as complex societies^3,4^. ASCs cause either a heightened awareness of the senses or the perception of alternate realities. How these states are achieved varies profusely between cultures, yet there are two principal pathways: the creation or activation of pathomorphic stressors (through, e.g., fasting, sleep deprivation, bloodletting), which lead to the liberation of endogenous opioid peptides (endorphins)^5^, and the administration of entheogenic substances of either plant or animal origin^6^. Either way, many societies have specialists, such as shamans, traditional healers, and doctors, who help community members in achieving ASC^7^. Moreover, McClenon^8^ argues the susceptibility to shamanic/hypnotic suggestion was positively selected in humans and their ancestors for the evolutionary benefits of pain reduction, enhanced healing, and easier childbirth.

Among entheogenic techniques, smoking in pipes is the most likely to be recognized archaeologically. However, although Pleistocene hunter-gatherers settling the Americas from Siberia arguably brought along a complex of shamanistic practices and medicinal plant use^9^, “nicotine delivery devices” (NDD) are an innovation not appearing in the North American archaeological record before the Late Archaic. The earliest known dates for the US southwest and northeast fall within the first millennium BCE^10^, while to this day the oldest finds from the Columbia River Basin reach back only 1200 years^11^. Tobacco (*Nicotiana* sp.) is the most prolific producer of nicotine^12^, but this metabolite has been detected in lower concentrations in other genera^13,14^. *Nicotiana* species also contain the psychoactive harmala alkaloids harman and norharman, which are known for their effects in drugs like *ayahuasca*^15^. Even though tobacco was widely documented as both a medicine and a sacred plant among Native Americans^16,17^, it was merely part of a much larger complex of psychoactive products, including plants that were smoked and that single-handedly account for dozens of genera^18^. Therefore, archaeological research of the co-evolution of humans and drugs requires thorough methodological foundations and state-of-the-art technological tools. In this paper, we present a metabolomics-based case study from Mesoamerica with a focus on tobacco preparations as a window into the modes of drug consumption.

In Mesoamerica, native communities such as the Nahua and Maya feature ritual specialists or shamans who communicate with supernatural entities and ancestors through ASCs induced by ingesting psychoactive plants as well as other pathways. De la Garza^19^ states tobacco is likely the most important sacred plant for the ritual and daily lives of Mesoamerican people. Interestingly, she specifically refers to *N. rustica* rather than the more commonly known *N. tabacum*, which forms the base of present-day commercial products. With access being socially restricted, ASC-inducing plant products were most likely kept in specialized containers in the past. De la Garza^19^ argues the *venenera* flasks of the Maya Area are a strong candidate for such artifacts. Although the vernacular term *venenera* translates to “venom bottle”, these vessels have also been said to contain poison, medicine, pigment, or snuff. Throughout this article, we follow Loughmiller-Cardinal and Zagorevski^20^ in not making a priori assumptions about vessel contents and apply the neutral term “miniature flasks”.

While information on storage containers is scarce, ethnohistoric sources from Mesoamerica provide more background on delivery devices and the mind-altering products themselves. Friar Bernardino de Sahagun was the first outside observer to give detail on indigenous drug consumption in what is today central Mexico. He describes smoking products among contact-period Nahua in the following words:

There are many ways of these canes, and they are made from many and diverse fragrant herbs, ground and mixed with each other, and filled and packed of roses, of fragrant spices, of the bitumen known as *chapuputli*, and of mushrooms, and of roses called *poyomalli*, or of *tlzyetl*, which is an herb.

Sahagún 1999 [1569]:574.

Based on ethnographic observation^21^, Mexican marigold (*Tagetes lucida*) figures notably among these tobacco mixture ingredients. In Mexico and Guatemala, this annual herb is commonly known for its role in ceremonies for the dead, which appear to have pre-Columbian roots^22^. However, present-day Huichol communities in western Mexico continue to smoke dried leaves and flowers either by themselves or in a mixture with *N. rustica*^21^.

Similar to most of their Mesoamerican neighbors, the ancient Maya did not use stone or clay pipes but other, more degradable NDDs such as reeds and corn husks^23^. However, smoking was not the only way tobacco was consumed by the indigenous groups of Mesoamerica. Goodman^24^ states chewing, drinking, snuffing, and enemas were all common forms of tobacco ingestion in the pre-Contact Americas. A contemporary of Sahagún’s *Historia General de las Cosas de Nueva España*, the *Descripción de San Bartolomé* (1588), for example, mentions chewing of pulverized tobacco mixed with lime by the Tz’utujil Maya of highland Guatemala. The preparation was recognized for its strengthening and thirst-quenching properties and kept in small gourds^25^. More recent ethnographic observations demonstrate the persistence of these consumption and storage patterns for tobacco-based products. Winter^26^ reports Mazatec of Oaxaca consistently carrying powdered green tobacco leaves in little gourds. The powder is used to relieve fatigue and in witchcraft. Similar practices are observed for the Huichol^27^ and the Tzotzil Maya of Chiapas who call the powder-lime mixture *mai*^28^, a term used for tobacco by several Maya sub-groups.

In an effort to trace tobacco consumption among the pre-Columbian Maya, Zagorevski and Loughmiller-Newman^29^ successfully identified nicotine residues in a ceramic miniature vessel using liquid chromatography–mass spectrometry (LC–MS) methods. Although there is no provenience information for the flask, the ceramic type situates it in the Southern Maya Lowlands and the Late Classic (AD 550-850) period. The container is of particular interest, because of a series of painted hieroglyphs reading *yotoot may—*the home of tobacco^30^. The authors point out that the lack of derivatives like cotinine, as well as the seemingly natural levels of nicotine enantiomer ratios, make the presence of thermo-altered tobacco unlikely. In other words, the vessel appears to have held a “fresh” tobacco product rather than its consumed remains^29^.

Based on the available ethnohistoric and ethnographic data from larger Mesoamerica, our working hypothesis states that some of the miniature flasks found in the Maya Area contained mixtures of lime (likely Ca[OH]_2_), tobacco (*Nicotiana* sp.), and other psychoactive and/or aromatic plants. The creation of an alkaline oral environment assists in the extraction and absorption of alkaloid substances^31^. The largely karstic substrate of the Yucatan Peninsula provides for an abundance of raw material and the ancient Maya are known for their advanced lime-based industries^32^. To draw support for our argument, we extracted residues from a collection of pre-Columbian Maya miniature vessels and analyzed them for their chemical composition in comparison to a suite of native plants through a LC–MS platform. Our main objectives include the following:

- Compare archaeological residues to modern plant extracts to increase the possibilities of identifying specific compounds through the application of a recently established ancient residue metabolomics methods^33^.
- Compare fresh tobacco samples to extracts obtained from cured leaf material to examine the impact of plant processing techniques.
- Compare results for curated to recently excavated, untreated vessels to control for the effects of cleansing procedures on the number of extractable residues.
- Compare results for distinct vessels regarding form and aperture diameter to control for possible functional differences among types of containers.

## Materials

### Archaeological ceramics

This study includes data from 14 miniature ceramics. We separate these flasks into two broad categories: recently excavated (untreated) vessels and previously curated vessels (which had been exposed to restorative treatments). Representing the vast majority (n = 12), the second group of flasks was excavated during a series of archaeological salvage projects taking place over the past 15 years in and around the city of Mérida, Yucatán, Mexico as part of the Proyecto Arqueológico Región de Mérida (PARME). Even though substantial evidence exists for both Preclassic and Postclassic occupations, the region reached its demographic peak during the Late Classic^34^. Cist burials from this period tend to display an array of grave goods which occasionally include the miniature ceramics under study^35^.

The remaining two untreated artifacts were excavated in 2016 at the site of Ucanha, which has been studied as part of the Proyecto Arqueológico Sacbé Ucí-Cansahcab (PASUC, known as UCRIP in English). Ucanha was first occupied in the Middle Preclassic (1000-350 BCE) and became a monumental center with over a thousand inhabitants in the Late Preclassic (350 BCE–AD 250) and the Late Classic^36,37^. Stone causeways connect it to Cansahcab (5 km to the east) and Kancab and Uci (5 km and 13 km to the west, respectively). Both flasks were found during excavations of Structure 65, a large residential platform with megalithic retaining walls, located about 250 m south of the Ucanha monumental core. Ceramic evidence suggests that the residents of Structure 65 were more prosperous than most households at Ucanha.

While it cannot be completely ruled out that vessels were touched bare-handed by a present-day tobacco consumer during the excavation process, the large majority of PARME and PASUC project members are non-smokers. Once recovered, artifacts were immediately sealed into labelled plastic bags. It can be expected that the curatorial treatments PARME vessels underwent before their exhibition resulted in the cleansing of exteriors from contaminants deposited during recovery. While exact treatment protocols are unknown, distilled water, water-alcohol solutions, acetone, and xylene are agents commonly used by restoration laboratories of the Instituto Nacional de Antropología e Historia (INAH). Further contamination is unlikely as conservators and laboratory staff wore sterile gloves when handling the artifacts. During exhibition at the Yucatan Regional Museum of Anthropology “Palacio Cantón” PARME vessels were placed in closed vitrines. Moreover, their comparatively small apertures drastically reduce the potential of airborne contamination. Since the exhibition was disassembled, the artifacts have only been removed from sealed containment for the purpose of residue extraction.

**Figure 1:**
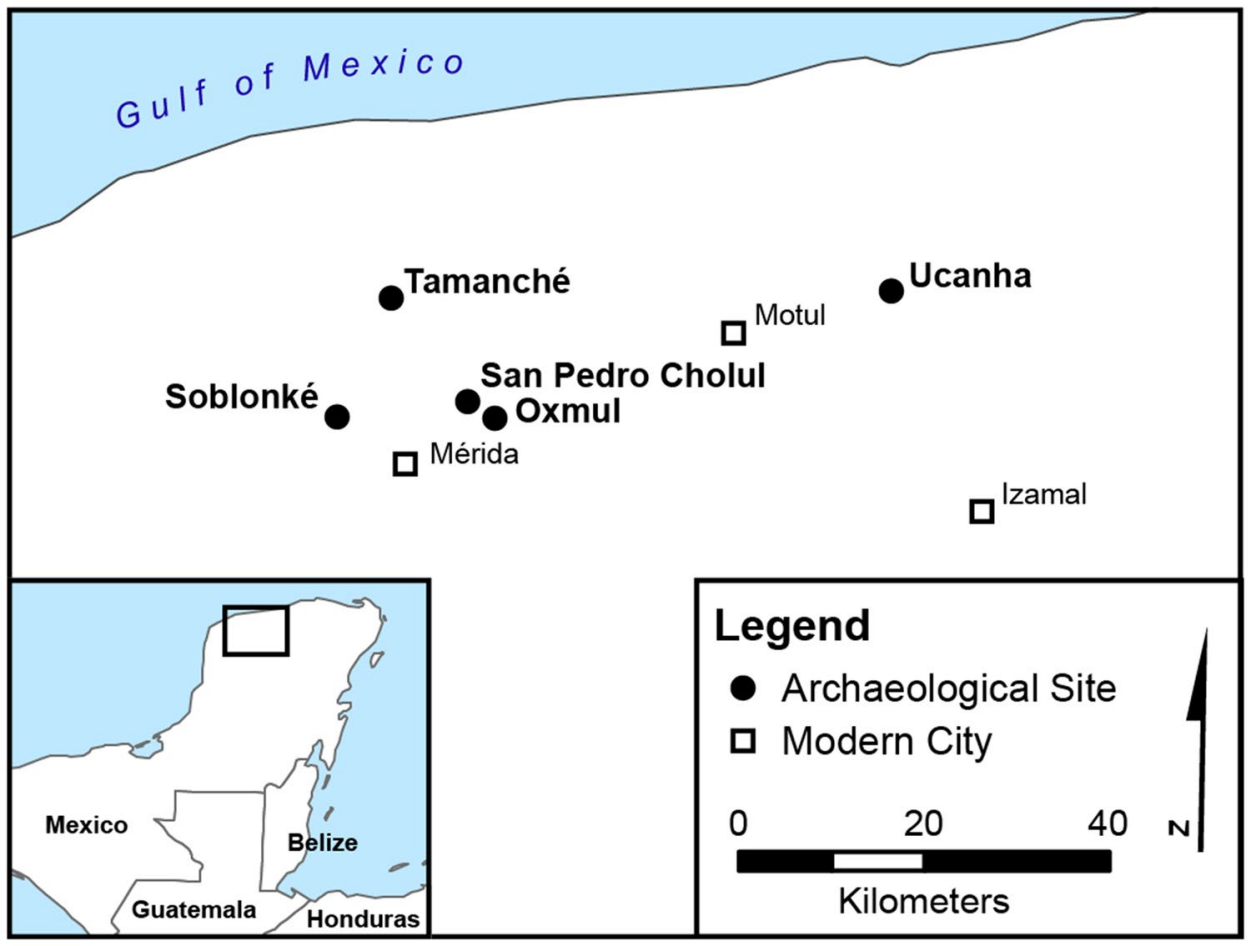
Map showing the archaeological sites of artifact recovery (generated via ArcGIS Desktop 10.7.1 [Environmental Systems Research Institute, 2020, Redlands, CA. https://desktop.arcgis.com/en/] using data from Natural Earth).

Based on typological analyses, most of the selected miniature vessels correspond to the Muna ceramic group and the Late to Terminal Classic transition (AD 750–900). One container belongs to the Late Classic Dzitya (AD 600–800) and another one to the Early Classic Aguila (AD 250–600) group. Following Loughmiller-Cardinal and Zagorevski^20^, the collection can be broken down into the following form classes: effigies with narrow apertures (n = 1), paneled flasks with narrow apertures (n = 9), sculpted flasks with narrow apertures (n = 2), and sculpted flasks with wide apertures (n = 2) (Fig. 1). By comparison, the unprovenienced flasks from the Kislak collection analyzed by Loughmiller-Cardinal and Zagorevski all display aperture diameters (2.9–3.2 cm) we would classify as wide. Similar to the Kislak vessels, PASUC and PARME sculpted flasks are consistently smaller in height and width compared to paneled ones. The latter frequently display applied buttons or serrated edges along their sides. While 60% of the Kislak vessels were designed to be strung as indicated by clay loops and wear marks around the neck, corresponding evidence is present in an even higher percentage (83%) in our collection (Table 1).

**Table 1:** PASUC and PARME vessels.

V#	Proj	Prov	Group	Period	Form	Decoration	Height (cm)	Width (cm)	Aper (cm)	Strung
1	PASUC	Ucanha	Muna	L–T	SF	None	5	5	0.7	Yes
2	PASUC	Ucanha	Muna	L–T	EF	Monkey	5.5	5.5	0.8	No
3	PARME	NE MID	Muna	L–T	PF	Serrated edges	6	7.1	0.8	Yes
4	PARME	NE MID	Muna	L–T	PF	Painted, Applied buttons	6.9	7.4	0.9	Yes
5	PARME	N MID	Muna	L–T	PF	None	6.6	7	0.9	Yes
6	PARME	N MID	Muna	L–T	PF	Painted	6.4	7	0.8	Yes
7	PARME	NE MID	Muna	L–T	PF	Serrated edges	5.8	6.2	1	Yes
8	PARME	NE MID	Muna	L–T	PF	Zoomorphic incision	8.2	9.3	1	Yes
9	PARME	NE MID	Muna	L–T	PF	Applied buttons, negative technique painting, incised	5.6	6.4	0.7	No
10	PARME	W MID	Muna	L–T	PF	Painted, applied buttons	7.1	7.8	0.9	Yes
11	PARME	NE MID	Muna	L–T	PF	Serrated edges	7.3	8.3	1.2	Yes
12	PARME	NE MID	Dzitya	L	SF	Painted, incised	4.8	4.7	1.1	Yes
13	PARME	NE MID	Aguila	E	SF	Incised	6.4	6.6	2.4	Yes
14	PARME	W MID	Muna	L–T	SF	Modeled owl face	5.3	5.4	2	Yes

*NE MID* Northeast of Mérida, *N MID* North of Mérida, *W MID* West of Mérida, *L–T* Late-Terminal Classic (AD 750–900), *L* Late Classic (AD 600–800), *E* Early Classic (AD 250–600), *PF* paneled flask, *SF* sculpted flask, *EF* effigy flask.

###  Reference species

As mentioned above, the reliance on relatively few biomarkers (i.e., anabasine, caffeine, nicotine, etc.) limits the ability to confidently determine which plant species are associated with which (type of) artifact. For instance, nicotine is present throughout the *Nicotiana* genus^12^. Hence, it does not allow for the discrimination of domesticated (*N. tabacum* [NT], *N. rustica* [NR]) from wild tobaccos (e.g., *N. attenuata*, *N. quadrivalvis*) by itself^11,33,38^. On the other hand, the continuing lack of compound libraries for LC–MS data requires a comparison of archaeological residues with substances produced by reference species in order to attain or approach species identifications. For this study, we selected a series of modern plant references based on the presence of psychoactive properties and/or their reported association with smoking, snuffing, or drinking practices in pre-Columbian Mesoamerica and the Greater Antilles (Table 2).

**Table 2:** List of reference species including information on human management and consumption.

Species	Code	Character	Distribution	Uses	Modes
*Nicotiana tabacum* (Tobacco)	NT	Domesticated	Across Americas	Medicinal, religious, stimulant (Winter 2000)	Smoking, Chewing, Snuffing, Drinking, Enemas (Goodman 1993)
*Nicotiana rustica* (Aztec tobacco)	NR	Domesticated	Across Americas	Medicinal, religious, stimulant (Winter 2000)	Smoking, Chewing, Snuffing, Drinking, Enemas (Goodman 1993)
*Tagetes lucida* (Mexican marigold)	TLU	Wild	Mexico, Central America	Aromatic, religious, recreational, medicinal (Siegel et al. 1977)	Smoking, Baths, Drinking (Siegel et al. 1977)
*Nymphaea* sp. (Water lily)	NSP	Wild	Across the world	Religious (de Rios 1974)	Drinking (de Rios 1974)
*Lonchocarpus violaceus* (Lilac tree)	LVI	Wild	Caribbean, Northern South America	Aromatic (de Lima 1990)	Drinking (de Lima 1990)
*Salvia divinorum* (Diviner’s sage)	SDI	Wild	Western Mexico	Religious (Valdés et al. 1983)	Smoking, Chewing, Drinking (Valdés et al. 1983)
*Datura wrightii* (Sacred datura)	DWR	Wild, Ornamental	Northern Mexico, Southwestern US	Medicinal (Stevenson 1915), Religious (Baker 2005)	Drinking, Chewing (Stevenson 1915)
*Anadenanthera peregrina* (Yopo)	APE	Wild	South America	Religious (Berenguer 1998)	Snuffing (Echeverría and Niemeyer 2013)

**Figure 2:**
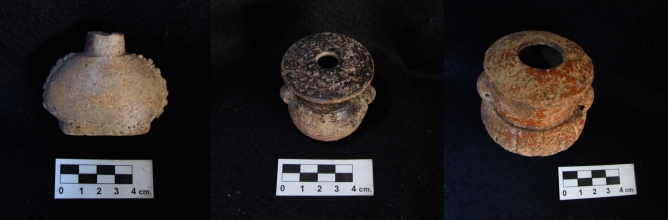
Miniature vessels: Paneled flask with narrow aperture (V11), Sculpted flask with narrow aperture (V12), Sculpted flask with wide aperture (V13).

Our hypotheses regarding the presence of these species in our archaeological samples vary. Even though terms for “tobacco” have been deciphered in Maya hieroglyphic writing and cigar smoking is occasionally represented in Maya art^39^, preserved plant remains are very scarce. Dedrick^40^ identified a single *Nicotiana* seed at the site of K’axob, Belize. As no wild tobacco species are native to the Yucatan Peninsula^41^ and morphological features resemble *N. tabacum* more than *N. rustica* (Dedrick, personal communication), it likely stems from the former. Morell-Hart and Cane^42–44^ recovered a total of seven specimens from four different sites in Honduras. The photography of the Los Naranjos find exhibits surface patterning again consistent with *N. tabacum*^42^ (Fig. 2). Withal, given their wide-spread distribution, we consider it to be very likely the pre-Columbian Maya had access to both species of domesticated tobacco. Assuming the vessels held mixtures of tobacco and aromatic ingredients, we consider Mexican marigold (TLU) to be a strong candidate for a complementary ingredient.

On the other hand, we disagree with the idea that waterlily (NSP) was used by the Maya to achieve ASCs. Hence, we do not expect to find chemical traces specific to that species. As *L. violaceus* bark is employed in alcoholic beverages rather than smoking, snuffing, or chewing mixtures, we also predict an absence of diagnostic compounds for the lilac tree (LVI). The situation is less clear for diviner’s sage (SDI), datura (DWR), and yopo (APE). Ethnohistorical and/or ethnobotanical sources attest to their consumption by neighboring ethnic groups. For yopo, in particular, its presence in the Greater Antilles has been evidenced archaeologically^45^. Given the long-distance trade networks the Maya were part of during the Classic period^46^, it is not unreasonable to infer the availability of such well-known intoxicating plants.

## Results

### Extracted features

**Figure 3:**
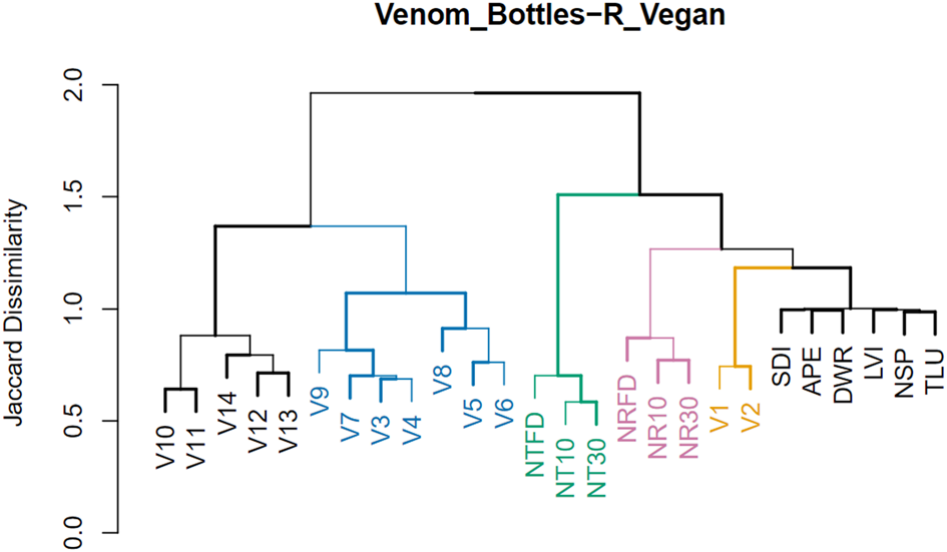
Clusters established by R Vegan applying Ward’s minimum variance method and the Jaccard index.

Our analysis yielded a list of 9092 mass spectral features in total from the combined collection of vessels and references analyzed. These chemical features represent compounds and adducts (and in some cases fragment ions) and isotopomers thereof. Initial cluster analyses (Fig. 3) yielded both expected as well as surprising results. Despite their different stages of curing (frozen, cured for 10 days, and cured for 30 days), the three sample types belonging to the two tobacco species group together (clusters in pink and green). The remaining modern references form a cluster of their own (in black at right). However, the PASUC vessels (in yellow) appear in between the latter and the *N. rustica* group on a branch which separates them completely from the PARME containers. No further relations based on spatial provenience are recognizable. On the other hand, vessel form seems to have at least some effect on recovered residues. The cluster formed by Vessels 03 to 09 (in blue) corresponds exclusively to paneled flasks with narrow apertures. The cluster of vessels including V10 through V14 (in black at left), however, contains three different forms of containers including sculpted flasks with wide apertures.

**Figure 4:**
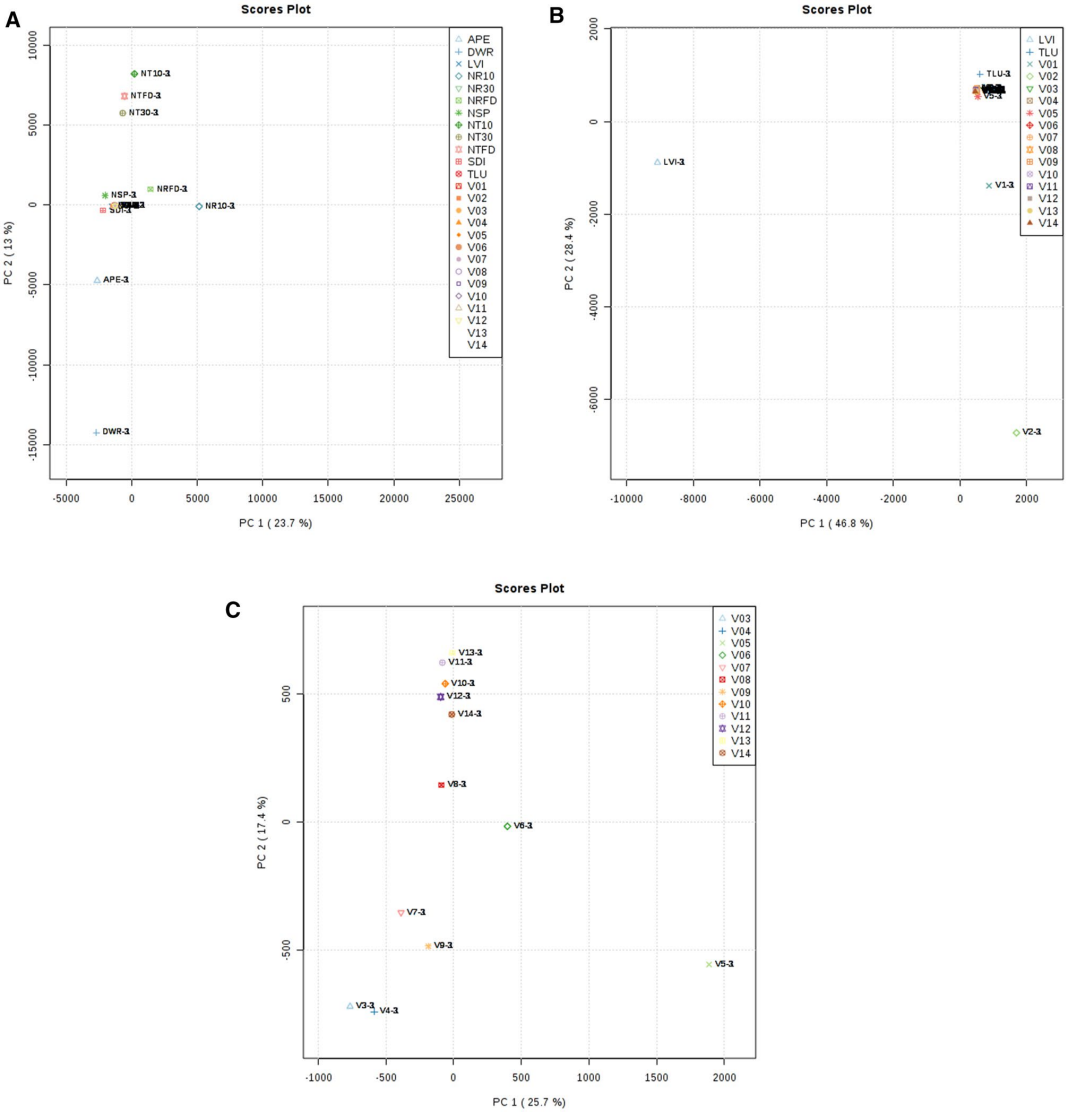
Principal components analysis (PCA) by MetaboAnalyst—(**A**) Vessels and all references; (**B**) Vessels, TLU, and LVI; (**C**) Vessels 03-14 only.

Further examination through successive principal components analysis (PCA) allowed for a more detailed perspective on the variance within the collection as well as similarity/distance between particular (groups of) samples (Fig. 4). Plot A highlights the distance both tobaccos as well as yopo and sacred datura keep with respect to the archaeological samples. This means when all 9092 features are considered, the archaeological samples appear to be more similar to the diviner’s sage and the locally collected plant references. Plot B, which only retains the two modern references most similar to the archaeological samples—Mexican marigold (TLU) and the lilac tree (LVI)— shows all archaeological samples scoring quite close to each other on Component 1. However, both PASUC vessels are more distant from their “peers” than TLU. This speaks to the degree of variation in residues extracted from curated versus untreated archaeological ceramics. Only after removing both remaining references as well as Vessels 01 and 02 is it possible to discern any meaningful patterns among the PARME vessels. Plot C essentially recreates the same clusters observed in Fig. 3 with the only notable difference being Vessel 05’s distance from the rest of the group.

**Figure 5:**
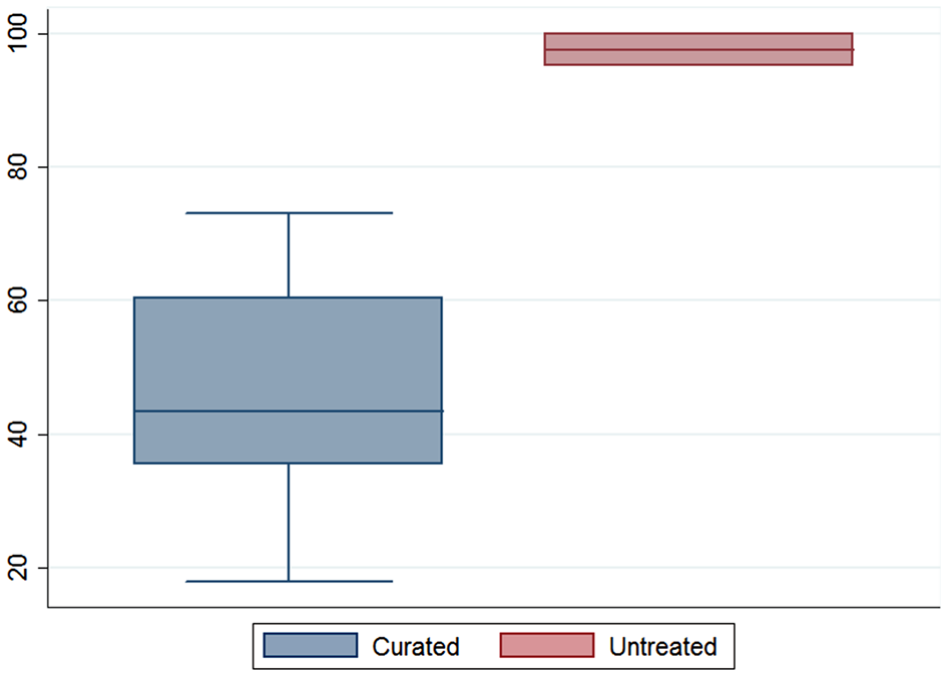
Box plot comparing the number of detected features between curated and untreated vessels.

The tight clustering of this larger group of artifacts seems to be directly related to the number of compounds reported per sample. While all modern references with the exception of LVI yielded more than 500 mass spectral features, Fig. 5 exhibits the noticeable quantitative difference between the archaeological vessels. While curated PARME flasks averaged 43 features, the untreated PASUC vessels more than doubled this mark yielding an average of 97 features. In other words, the lower number of recovered residues among the PARME vessels decreases the discriminatory potential of exploratory statistics such as PCA significantly. This panorama of homogeneity is likely a case of false negatives.

### Shared unique compounds

The examination of unique compounds found in our reference samples and shared by one or more of the archaeological samples has largely been unsatisfactory. First, we are unable to report a single occurrence of one of the four chemical standards run with the archaeological samples. In other words, we were unsuccessful in repeating the identification of nicotine made by Zagorevski and Loughmiller-Newman^29^ on a similar artifact, despite using instruments of at least comparable (LC–MS) or better (GC-TOFMS) sensitivity. Second, the frequency with which we were able to spot unique compounds in the archaeological samples was generally low. Features unique to SDI, NSP, and LVI were not detected in any of the archaeological samples. Overall, Vessel 13 displays the highest percentage of shared unique compounds: its extracts contained 4 out of 128 compounds exclusively identified in Mexican marigold (Table 3).

**Table 3:** List of unique compounds and their occurrence in archaeological samples.

Species	NT10	NR10	TLU				DWR	APE
Metab ID	6956	4225	8738	8776	8791	8813	1739	809
*m/z*	358.3	349.2	256.3	270.3	312.3	151.0	337.2	339.2
Ret time	11.97	10.22	11.43	11.49	11.38	0.82	11.29	11.71
V01	X				X			
V02	X				X		X	
V03	X		X		X			
V04			X					
V05	X							
V06	X		X					
V07								
V08	X		X	X				
V09	X		X			X		
V10			X			X		
V11			X			X		
V12			X			X		
V13		X	X	X	X	X		X
V14			X			X		

Nevertheless, when observing Table 3 several interesting patterns emerge. The ubiquity of mass spectral feature #6956 is remarkable. This single unique compound for *N. tabacum*, detected exclusively in leaves cured for 10 days, is shared by half of the archaeological samples. *N. rustica* is also only represented by a single unique compound extracted from the 10-day cured sample (#4225). However, this compound is shared by only one vessel. Together, the NT10 and NR10 samples indicate that although the tobacco references are distant from the archaeological samples when all features are considered, specific signals might be shared consistently. The highest overall ubiquity value corresponds to a Mexican marigold compound (#8738). This unique compound is shared by 10 of the 14 archaeological samples. Aside from the mass spectral features #6956, #8738, and #8813 (also associated with TLU), the remaining unique compounds fail to be shared by more than 4 out of 14 vessels.

Finally, we did not identify any of the shared unique compounds detected in this study; a situation not uncommon in non-targeted metabolomics-based analyses. Even though relatively low mass error ranges for *m/z* peaks allowed us to reconstruct potential molecular formulas for several of those compounds, none of the potential molecules appeared to be present in the KNApSAcK database^47^.

## Discussion

Although being only the second study of this type, the patterns emerging from the application of ancient residue metabolomics established by Brownstein et al.^33^ are relevant for future research regarding specific molecular residue signatures in ancient ceramics. By focusing on the entire set of extractable compounds, rather than a pre-selected suite of biomarkers, we were able to observe that post-recovery treatment does impact chemical residue datasets significantly. Restorative practices, directed towards the cleansing and recovery of surface features such as paintings or reliefs, clearly reduce the compounds available for extraction. This fact is likely due to the use of diverse solvents during these procedures.

By comparison, the lack of association based on spatial provenience in our dataset is encouraging. In other words, the soil matrix surrounding the vessels after deposition seems to have less of an impact on the results than their contents. The PASUC vessels strongly support this argument as their metabolomic variance is significantly greater when compared to their PARME counterparts, even as the former were recovered from a single structure, while the latter belong to several sites in a larger region. Miniature vessels such as the ones we analyzed most likely held different products depending on their general form and, particularly, the size of their apertures. We observed similarity specifically among paneled flasks with narrow apertures.

The analytical work on shared unique compounds, an approach most similar to the GC–MS focus on specific biomarkers, led to additional important conclusions. The first concerns the characterization and analysis of modern references, which needs to incorporate a larger array of processed products of those candidate species for ancient residues. Humans do not often consume plants or animals in a raw state. It is much more likely for organic products to be somehow manipulated before serving their final purpose. The exclusive presence of unique compounds from cured rather than fresh tobacco leaves in our archaeological samples points in this direction. The second remark pertains to the type of species selected for references. Although our working hypothesis led us to focus on tobaccos and their derivative compounds, the analyses showed us that more evidence might emerge regarding secondary ingredients such as Mexican marigold (*T. lucida*), an aromatic additive in tobacco mixtures reported by Colonial writers like Friar Bernardino de Sahagún^48^. In our study, chemical features unique to *T. lucida* were more commonly detected in archaeological samples than any other targeted substance including the biomarker nicotine. When all signals were considered for analysis, the flasks grouped closer to TLU compared to either NT or NR. This is the first time that Mexican marigold has been detected in archaeological residues, and its association with miniature ceramics contributes to an increasingly nuanced paleoethnobotanical record for Mesoamerica. On the other hand, evidence for the storage of material originating from water lily, lilac tree, diviner’s sage, or yopo is either inconsistent or absent.

The presence of residues associated with Mexican marigold is illuminating for the archaeological study of human use of drugs and cultural practices related to ASCs in general. It provides insight into the persistence in use of certain substances that have maintained importance among native communities in the region. Like other indigenous American cultures, Mesoamerican people like the Nahua or Maya appear to have mixed psychoactive substances with other products^21^. Almost a century ago, Miner^31^ called attention to the cross-cultural parallels in the use of lime to enhance the effects of alkaloids contained in tobacco, coca (*Erythroxylum coca*), or betel (*Piper betle*). Adams et al.^49^ suggest tobacco might have been placed inside of *Yucca* quids in the Southwestern US to reduce gingival irritation or to more evenly spread nicotine release over a longer period of chewing. Siegel et al.^21^ report that Mexican marigold was used because it “reduces the harshness of *yé* [*N. rustica*]”.

In conjunction with ethnohistoric reports of tobacco mixtures, these observations indicate the paraphernalia surrounding drug consumption extends beyond tools for preparation, delivery devices, and containers. A holistic understanding of entheogenic drug complexes ought to incorporate the non-psychoactive ingredients of the ingested products. Further interdisciplinary research is necessary to first detect these elements and then identify their roles in achieving ASCs. Biomolecular archaeology and ancient residue metabolomics clearly can add significantly to such research and discussions about the role of psychoactive plants throughout the world.

## Methods

### Modern plant references

NT and DWR plants were grown in WSU greenhouses from seed acquired through Horizon Herbs Strictly Medicinal (Williams, OR, USA). Similarly, NR seeds were acquired through the United States Department of Agriculture (USDA) Agricultural Research Services (ARS) National Plant Germplasm System (NPGS) (PI 555554). SDI cuttings were originally taken in the Sierra Mazateca mountains of Oaxaca, Mexico, and then also propagated in WSU greenhouses. APE seeds were ordered through Natural Ether (Coral Springs, FL, USA) and ground in order to simulate the reported snuffing mode of administration (e.g., Ogalde et al. 2009). TLU leaves and LVI bark shavings were collected on the Campus de Ciencias Sociales, Económico-Administrativas y Humanidades of the Universidad Autónoma de Yucatán (UADY), Mexico and NSP flowers and stalk material on the Campus de Ciencias Biológicas y Agropecuarias, also of UADY, Mexico.

The preparation of reference samples proceeded along the following protocol: With the exception of both *Nicotiana* representatives, one sample was prepared for each candidate species using dried leaves (TLU, SDI), stem and flower (NSP), rind (LVI), or ground seeds (DWR, APE). For both tobaccos, a sample of freeze-dried leaves (NTFD; NRFD) was compared to material cured for 10 (NT10; NR10) and 30 days (NT30; NR30), respectively. In addition to references created directly from modern plants, chemical standards for LC–MS analysis of nicotine, as well as atropine, tropine, and scopolamine, which are found in *Datura* species, were purchased from Sigma-Aldrich (St. Louis, MO, USA).

### Extraction and chromatography

Vessels were emptied of deposited sediment in advance of residue extraction for metabolomic analysis. Disposable wooden sticks were used to pry dry soil from flask interiors. The resulting samples were used for semi-quantitative chemical tests and macrobotanical analysis, which did not yield evidence for tobacco or other plant species^50^. The extraction protocol included the successive exposure of the interior surfaces of all 14 vessels to three different solvent systems: (1) 2% aqueous tartaric acid (TA), (2) acetonitrile:2-propanol:water [3:2:2] (APW), and methyl *tert*-butyl ether (MTBE). We followed this approach to increase our ability of capturing compounds with different polarity characteristics. Each vessel was filled with 10 ml of TA and then sonicated for 10 min. Afterwards, the supernatant was transferred to a clean vial and the extraction was repeated with the other two solvents, APW second and MTBE last. Sonication has proven to consistently extract chemical residues from the matrix of artifacts made from clay and porous rocks^51^, thereby preventing the reliance on samples originating from surface layers which have been shown to be susceptible to airborne contamination^52^. Sonication also eliminates the need for supplies used to clean or abrade artifact surfaces such as cotton balls or sandpaper, which have similarly been acknowledged as vectors of contamination^53^. Nonetheless, to control for possible intrusive substances, blank samples were created for all three solvents. Blanks were collected in the same type of vial used for the archaeological samples and from thereon processed identically. This procedure guarantees that all possible contamination stemming from supplies or instruments can be accounted for during data processing.

Depending on the solvent system, different techniques were then applied to convert extracts to a solid state: TA samples were freeze-dried; APW samples rota-evaporated and freeze-dried; and MTBE samples air-dried in a fume hood. The protocol was identical for the modern plant references. In addition, blank samples were run for each solvent. All solvents used for extraction and analyses were of liquid chromatography-mass spectrometry grade. Resuspension occurred following the protocol previously employed by Brownstein et al.^33^. We used 0.6 ml of 0.1% formic acid/water:acetonitrile [1:1] for TA and APW extracts and an identical volume of 0.10% formic acid/acetonitrile for MTBE extracts. Resuspended extracts were vortexed and centrifuged at 10,000×*g* for 10 min at 4 °C, before a 0.5 ml aliquot was transferred to liquid chromatography sample vials.

Similarly, instrument configuration resembled the setting published by Brownstein et al.^33^. Ultra-performance liquid chromatography (UPLC) was conducted on a Waters ACQUITY UPLC system (Waters Corporation, Milford, MA, USA) with photodiode array (PDA) detection ranging between 210 and 400 nm followed by inline mass spectrometric analysis. One microliter of sample was injected through a 2.0 µL sample loop using the full loop injection mode, and the flow rate through a Waters ACQUITY UPLC T3 column (HSS T3, 1.8 µm, 2.1 × 100 mm) was 0.32 mL/min with 0.10% formic acid/water (A) and 0.10% formic acid/methanol:acetonitrile [2:3] (B) in a slightly concave gradient elution mode. The gradient elution was applied as follows—97% A:3% B to 15% A:85% B from 0.00 to 12.00 min, changed to 3% A:97% B in 0.10 (12.10) min, maintained at 3% A:97% B until 14.00 min, returned to the initial conditions of 97% A:3% B in 0.10 (14.10) min, and then, before the next injection, maintained at 97% A:3% B until 16.00 min. The analysis time was 16.00 min. The autosampler chamber and column temperature were 8 °C and 35 °C, respectively.

A Waters SYNAPT G2-S HDMS Q-TOF with lockspray ionization was operated in ESI positive and resolution mode (mass resolution of ~ 40,000 for the compound range analyzed). The scan range was from 100 to 1200 *m/z* with a scan time of 0.3 s. Mass spectral data were collected in profile mode using MS^E^ with high collision energy (ramp 15–40 V) for fragmentation. The capillary voltage, sampling cone voltage, and source offset voltage were 3.0 kV, 60 V, and 60 V, respectively. The source temperature was 100 °C with a cone gas (nitrogen) flow rate of 50 L h^−1^. The desolvation temperature was 250 °C with a desolvation gas (nitrogen) flow rate of 900 L h^−1^. The nebulizer gas (nitrogen) flow was 6.0 bar and the lock mass compound was leucine enkephalin with a reference mass of 556.2771 [M + H]^+^ *m/z*^33^.

### Data processing

Although Waters LC–MS systems are regularly used in association with Progenesis QI software, our team extended data processing and multivariate statistical analyses to the application of a series of different open-source tools including MZmine 2^54^, MetaboAnalyst^55^, Draw Venn Diagram^56^, and the “vegan” package for R^57^. The detailed data processing method is described in Brownstein et al.^33^. However, before carrying out any statistical procedure, the list of mass spectral features was reduced by all compounds encountered in solvent blanks. All datasets were submitted to the MetaboLights^58^ repository (study identifier: MTBLS2018).
